# Imaging, Surgical, and Pathology Findings of a Myxoid Aortic Sarcoma: When Distal Embolization Uncovers a Bigger Problem

**DOI:** 10.1055/s-0039-1678548

**Published:** 2019-02-15

**Authors:** Domenico Calcaterra, Carlo M. Rosati, Leslie A. Renfro, Panayotis N. Vardas

**Affiliations:** 1 Department of Anesthesiology, Minneapolis Heart Institute Foundation at Abbott Northwestern Hospital, University of Minnesota Medical School, Minneapolis, Minnesota; 2 Division Cardiac Surgery, Indiana University, Indianapolis, Indiana

**Keywords:** aortic sarcoma, distal embolization, imaging, surgical resection, adjuvant therapy

## Abstract

Aortic sarcomas are a very rare condition typically characterized by a deceiving presentation. Making a correct diagnosis is based on the application of an algorithm which allows to identify the primary disease site and to obtain a tissue diagnosis. Surgical aortic resection with adjuvant therapy offers the best palliation, particularly in cases of well-differentiated tumors with no evidence of diffuse metastatic spread.

## Case Presentation

**Fig. 1:**
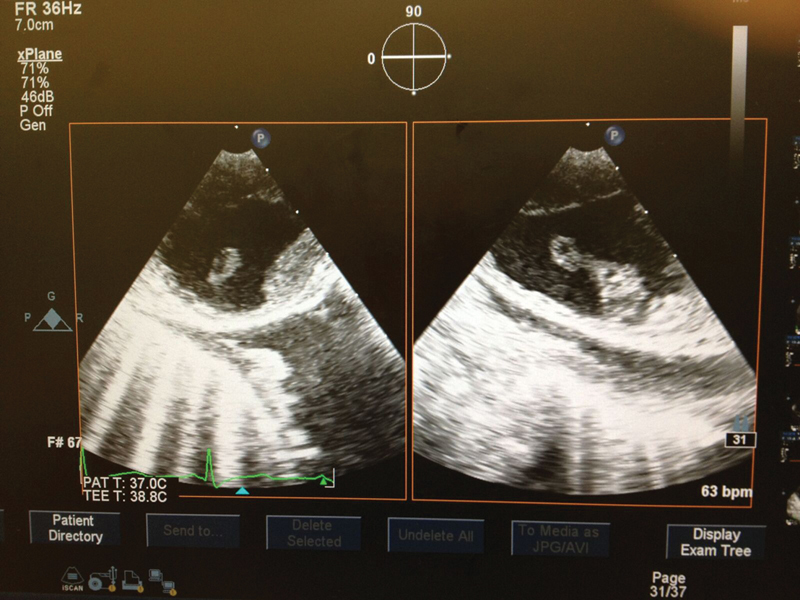
Echocardiography images of the descending thoracic aorta showing intraluminal floating mass.

**Fig. 2:**
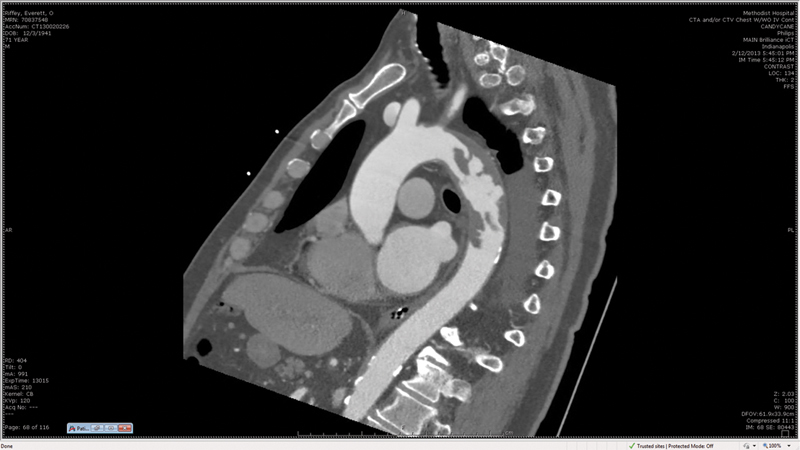
Computed tomography scan image.

**Fig. 3:**
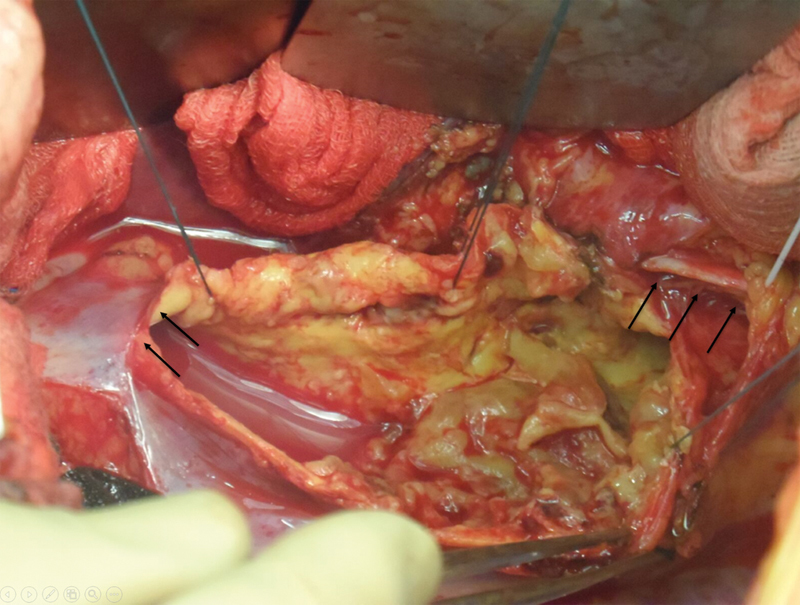
Intraoperative findings: descending thoracic aorta opened longitudinally (arrows to the left point to the distal descending thoracic aorta and arrows to the right show the left recurrent laryngeal nerve).

**Fig. 4:**
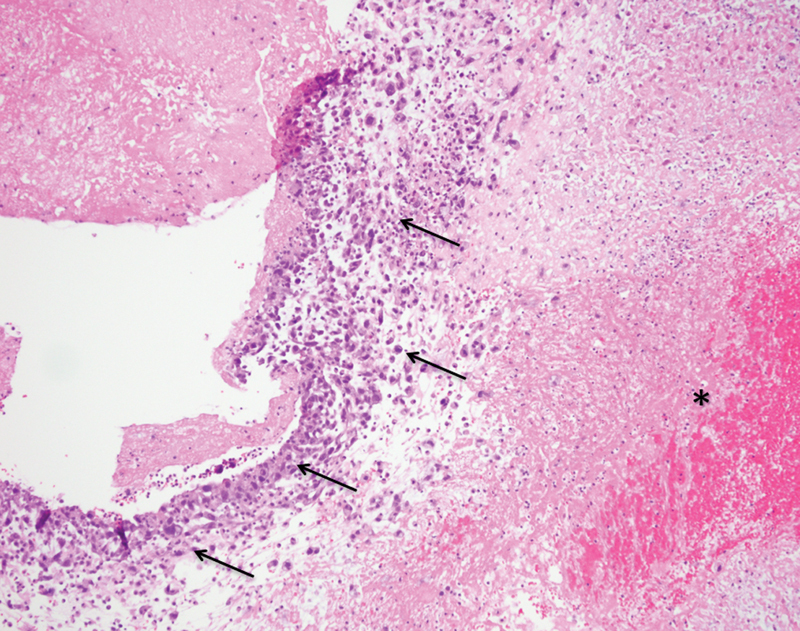
Histologic exam. Arrows show malignant tumor cells within intraluminal aortic mass (asterisk) (20 X).

**Fig. 5:**
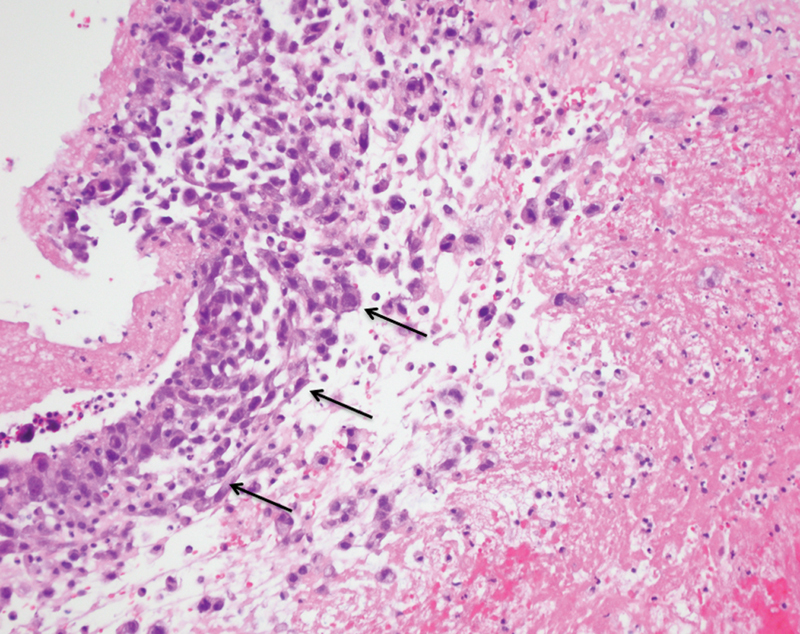
Histologic exam. High power view (40 X).

A 71-year-old male, transferred from a rural hospital after an emergent small bowel resection, was diagnosed with a floating aortic mass involving the descending thoracic aorta (DTA) ( Figs. 1 and 2 ). Idiopathic thoracic aortic thrombosis was considered as the most likely primary diagnosis. The patient underwent replacement of the DTA with full cardiopulmonary bypass under deep hypothermic circulatory arrest. Access was established through a left thoracoabdominal incision with inflow cannulation of the left carotid and left femoral artery and outflow cannulation of the left femoral vein. At time of surgery, the aorta was found to be filled by a gelatinous material ( Fig. 3 ). The specimen analysis showed a poorly differentiated mucinous tumor, characterized as a high-grade myxoid intimal aortic sarcoma ( Figs. 4 and 5 ). The patient had a slow postoperative recovery without cerebrovascular accidents. Nonetheless, within 3 weeks of surgery, he was diagnosed with diffuse metastatic liver disease. This diagnosis and the overall rapid decline in his health conditions precluded any further adjuvant treatment and he was transferred to hospice care.

## Discussion

Primary aortic sarcomas are a very rare condition which carries a dismal prognosis. 1 They include leiomyosarcoma, fibrosarcoma, angiosarcoma, hemangioendothelioma, and myxoid sarcoma. 1 They are more commonly localized in the abdominal aorta and they arise from the aortic media or the intima, with the latter being the most common. 2 Among aortic sarcomas, myxoid type is almost a unique finding. 3 Intimal sarcomas typically present with symptoms of distal embolization, whereas sarcomas arising from the media have an even subtler presentation. 2

The challenge of establishing a correct diagnosis in cases presenting with distal embolization relies on the implementation of an algorithm aimed at identifying the source of embolization and defining the nature of the primary pathology. Once the heart is ruled out as the primary possible embolization site with transesophageal echocardiogram, evaluation of the aorta by computed tomography scan or magnetic resonance imaging is required, 4 5 since a primary pathology of the aorta such as an intraluminal wall thrombus and an infectious or neoplastic process can represent the embolic source. Differential diagnosis includes, in absence of aneurysmal disease or severe atherosclerosis of the aorta, idiopathic thoracic aortic thrombosis, aortitis, and a neoplastic or infectious process. 4

Diagnosis is obtained by microscopic examination of the secondary embolization site and by pathology examination of the primary aortic specimen.

Indication for aortic replacement is represented by any floating aortic mass associated with distal embolization. 4 5 Also, the need to obtain a tissue diagnosis may indicate surgery. In the case presented, the choice for left carotid artery cannulation as inflow for cardiopulmonary bypass with deep hypothermic circulatory arrest was dictated by the need to avoid retrograde aortic flow and aortic cross-clamping to minimize the risk of cerebrovascular embolization.

In cases of aortic sarcoma, aortic replacement with adjuvant therapy offers the best palliation. Longer survival is achieved for differentiated tumors but surgery may be futile in cases with high tumor grade and diffuse metastatic spread. 3
